# A High Enrichment Replenishment Rate Reduces Damaging Behaviors and Increases Growth Rate in Undocked Pigs Kept in Fully Slatted Pens

**DOI:** 10.3389/fvets.2020.584706

**Published:** 2020-11-13

**Authors:** Jen-Yun Chou, Dale A. Sandercock, Rick B. D'Eath, Keelin O'Driscoll

**Affiliations:** ^1^Pig Development Department, Teagasc, Animal & Grassland Research and Innovation Centre, Moorepark, Fermoy, Ireland; ^2^Animal & Veterinary Sciences Research Group, SRUC, Roslin Institute Building, Easter Bush, Midlothian, United Kingdom; ^3^Royal (Dick) School of Veterinary Studies, University of Edinburgh, Easter Bush, Midlothian, United Kingdom

**Keywords:** growing-finishing pigs, point-source enrichment, grass, tail biting, tail docking, harmful behaviors, pig production

## Abstract

One of the difficulties in complying with the prohibition of routine tail docking is a lack of effective alternative solutions to prevent tail biting, especially in fully slatted systems. This study compared three slat-compatible enrichment replenishment strategies for pigs. Forty-eight mixed-sex pens (six males and six females/pen) of undocked pigs were followed from birth to slaughter. Pre-weaning, half the pigs were provided with enrichment materials (a cardboard cup, rubber toy, hessian cloth and bamboo), in addition to a rope for the sows, in all farrowing crates. Post-weaning, all pens were enriched with eight identical items, including an elevated rack supplied with fresh-cut grass, and objects of wooden, bamboo, rubber, and fabric materials presented in various ways. However, three different replenishment frequencies were applied: “Low” (replenished on Monday/Wednesday/Friday), “Medium” (replenished once daily), and “High” (replenished *ad libitum*). Individual pigs were weighed on days 0, 49, 91, and 113 post-weaning. Direct behavior observations were conducted twice weekly at pen level (10 min/day/pen), and tail and ear lesion scores of individual pigs were also recorded every other week. These measurements were taken during the post-weaning period. The cost of all enrichment materials used was calculated. Pre-weaning enrichment only contributed to a lower ear lesion score (*P* = 0.04). No difference in lesion scores was found between post-weaning treatments. “Low” replenishment rate pigs performed more damaging behaviors (tail/ear biting, belly-nosing, mounting, other biting, and aggressive behaviors combined) than “High” and “Medium” pigs (*P* < 0.01). The average daily gain in the finishing stage was higher in “High” than “Low” pigs (*P* < 0.05). Although sporadic tail biting occurred, only 0.69% of the pigs had their tails bitten severely enough that they became shorter than half of a normal undocked tail. The average enrichment cost for the post-weaning period was <€2 per pig. In conclusion, the high enrichment replenishment rate increased growth and reduced damaging behaviors compared to the low replenishment rate pigs. Overall, these findings show that the provision and regular replenishment of multiple, slat-compatible, enrichment sources can reduce tail damage to manageable levels without the need for tail docking.

## Introduction

Although it has been more than a decade since the ban on routine tail docking to control tail biting was codified into Council Directive 2008/120/EC (1), the search for solutions to manage tail biting and to implement the non-docking policy is still on-going. The reason why this policy was not readily implemented is due to a multitude of factors, including the unpredictability of tail biting, its multifactorial nature, difficulty in its management, reluctance to change current rearing practices, and increased production costs associated with rearing undocked pigs (2). Tail docking is also still commonly practiced because it does reduce the risk of tail biting, although not completely. A study found more frequent tail biting behaviors and worse tail lesions when pigs were not docked compared to their docked counterparts in the same herd with identical management practices (3). However, even among docked pigs, tail lesions can still be observed in slaughterhouse inspections (4), which suggests that docking only reduces the severity of tail biting rather than preventing it entirely.

Other than docking status, another important risk factor for tail biting is the availability of adequate manipulative materials as environmental enrichment. Effective enrichment should be able to stimulate species-specific behaviors and prevent damaging behaviors, for example, tail biting is considered a redirected foraging behavior (5). Thus, enrichment should allow pigs to investigate, manipulate, chew and ingest (6), and sustain their interest. Provision of loose bedding materials such as straw satisfies these criteria and is considered effective in reducing tail biting (7–9), but on fully slatted floors straw can block slurry removal systems (10). Many studies have investigated alternative ways of supplying loose materials that are compatible with slatted floors: in elevated fittings (11–16), as a compressed form in solid blocks (16, 17), or in floor feeders (18). However, simply providing loose materials in a fixed location for pigs to interact with has not been as effective in reducing tail biting in undocked pigs as provision of material on the pen floor (14, 18); what is more effective when managing tail biting in undocked pigs, is combining the provision of loose materials in a smaller quantity with other point-source enrichment items (13, 19).

Although the benefit of enrichment has been widely acknowledged among stakeholders (20), the awareness of its importance and the uptake and use of suitable enrichment materials in commercial practices in the EU has still been low (6, 20). A significant obstacle is the perceived negative economic impact on production costs, in terms of the supply of the actual materials and the extra labor required for maintenance (21). In order to reduce these costs, some producers use objects that are either of inappropriate materials or presentation (e.g., a hanging metal chain), which may lead to negative effects on pig welfare (6). On the other hand, the benefit of appropriate enrichment provision on pigs' performance is well documented (22, 23). An optimal enrichment solution will need to strike a balance between allowing undocked pigs to thrive in their environment and minimizing labor and cost. Research is needed to identify this balancing point and determine whether the costs associated with rearing undocked pigs with appropriate enrichment provision can be offset by benefits to the pigs' health, performance and carcass traits.

Beside the rearing environment during the growing and finishing stages, there is some evidence that shows the pre-weaning environment has an impact on the risk of tail biting. Weaned pigs, which were housed on partly-slatted floors with a single rubber hanging ‘toy', performed fewer pig-directed manipulative behaviors if they had been provided with an enriched environment pre-weaning, than if the environment was barren (24). However, if pigs were housed in straw-bedded pens post-weaning, there was no effect of pre-weaning environment on the frequency of harmful behaviors. Likewise, Telkanranta et al. (25) found pigs performed fewer manipulative behaviors with the snout or mouth directed toward other pigs, and had a lower percentage of severe or mild tail lesion 5 weeks post-weaning, when they had been housed in a more enriched environment (rope, newspaper, ball, and wood shavings compared with only ball and wood shavings) pre-weaning. Other studies reported no difference in terms of post-weaning tail biting behaviors or tail lesions between early-enriched (with substrates such as straw, peat, and wood shavings) and barren housed pigs (26, 27). Thus, further investigation is needed to understand how pigs' early life experience with enrichment can affect their predisposition for performing damaging behaviors later in life, especially in fully slatted floor systems.

This study employed a 2 × 3 design to compare enrichment strategies: pre-weaning exposure to environmental enrichment or not, and three enrichment replenishment rates post-weaning. All enrichment materials used from farrow to finish were compatible with fully slatted systems and, based on a previous study, were shown to be biologically relevant to satisfy pigs' explorative behaviors (13). It was hypothesized that early exposure to an enriched environment and a high enrichment replenishment rate would reduce tail biting behavior and tail lesions. An additional aim was to also calculate the economic costs and benefits of this complex enrichment strategy for use with undocked pigs on this type of flooring system.

## Materials and Methods

All procedures in the study were approved by the Teagasc Animal Ethics Committee (TAEC163-2017). Due to the ethical concern of severe tail biting events that might hamper pig welfare, all pigs were checked 3 times daily by the experimenter and additionally by the farm staff for any signs of tail biting, following a previously published protocol on tail biting outbreak intervention (28). In short, three intervention methods were used: removing biters, removing victims and providing additional enrichment (three ropes). The intervention methods were deployed randomly one by one regardless of the treatment groups until the outbreak was controlled. A detailed description and outcome of the intervention protocol can be found in Chou et al. (28). The individual animal identification and details of the causes of any temporary removals or medical treatments applied to pigs, either due to tail biting or other health issues, were recorded. The previous study found no difference of the intervention methods in the effectiveness of halting tail biting outbreak or reducing tail lesions (28), and therefore did not interfere with this main study. In reality, tail biting outbreak intervention is essential when pig producers rear undocked pigs as there is always a risk of tail biting (29–31); thus, we fully incorporated the interventions while conducting this study. The number of outbreaks and temporary removals of pigs were evaluated between treatments as an additional indicator of the severity of tail biting events.

### Animals and Housing

A total of 576 pigs, born over five batches to 59 sows in the Teagasc Moorepark Research unit (Ireland), were used in the study. A 3-week batch farrowing system was practiced, and therefore piglets in batches one to four were born 3 weeks apart (15 sows in batch 1; 10 sows in batch 2; 14 sows in batch 3 and 4). The fifth batch (6 sows) farrowed 15 weeks after the fourth batch and was used to compensate for a lower than expected number of animals available in batch 2, to reach the targeted sample size. Piglets were teeth-clipped at 2 days of age to prevent excessive damage to their faces and the sows' udders. The pigs' tails were left undocked, and male pigs were not castrated. All piglets were tagged on the left ear for individual identification 24 h after birth. The sow and piglets were housed in a conventional farrowing pen (2.4 m × 1.8 m), including a metal farrowing crate (2.2 m × 0.6 m) for the sow, and a floor heating plate (1.6 m × 0.4 m) for the piglets. A nipple drinker for the piglets was provided on one side of the pen wall, and a synthetic hemp rope (1.2 m hung from one side of the crate) was provided to the sow as environmental enrichment. The temperature was kept at 24°C after farrowing with a heat plate to maintain piglets' thermal comfort. The farrowing pens had fully slatted floors except the heating plate area. Creep feed was provided in a floor feeder starting from 3 weeks of age.

Piglets were weaned on the same day at around 4 weeks of age. Two days before weaning, all pigs were individually weighed and randomly allocated to their post-weaning treatment (12 pigs/pen). Allocation was balanced for pre-weaning treatment, weight, sex (six male and six female) and litter mates (with 2–4 litter mates together). Piglets with open wounds on the tail and lower than 4 kg body weight were not selected (2/7/4/3/7 pigs respectively in each batch). At weaning, all piglets were transported by a wheelbarrow to the weaner accommodation and mixed into their treatment groups. A standard pelleted diet was provided *ad libitum* by a wet-dry feeding system consisting of single space feeders. Water was also provided *ad libitum* in a separate nipple drinker. During the first week post-weaning, a starter diet was provided (Startrite 88, Provimi, Ireland), then a standard home-milled link diet for 2 weeks, before a standard commercial weaner diet was provided (net energy 10.99/9.67 MJ/kg, protein 17.9%/16.18%, crude fiber 3.3%/5.06% for weaners and finishers respectively).

Weaner pens were 2.4 m × 2.6 m in dimension with fully slatted plastic floors. At 7 weeks post-weaning (i.e., 11 weeks of age) pigs remained in their groups of 12 and were transferred to finishing pens (4.0 m × 2.4 m) with fully slatted concrete floors, where they remained for another 9 weeks until the end of the study (20 weeks of age). The temperature was maintained at 27–28°C immediately post-weaning by a computer-controlled heating and mechanical ventilation and reduced 2 degrees every 2 weeks thereafter in the weaner house. In the finisher house the temperature was kept at 20°C by a computer-controlled mechanical ventilation system. Heat produced by the pigs meant this was achieved without an artificial heat source. Artificial lighting (around 150 lux and 130 lux in the weaner and finisher house respectively) was provided between 0,700 h and 1,700 h to supplement natural light from windows along the walls of all rooms and promote a normal circadian rhythm.

### Experimental Design

The study used a 2 × 3 factorial design: enriched or barren environment pre-weaning and three different enrichment replenishment rates post-weaning. We did not use a “no enrichment” negative control treatment as it is illegal in the EU, and based on the research team's past experience, it would only lead to severe tail biting and constant removal of pigs from the study without a meaningful justification.

One week after birth, litters included in the study were allocated to two pre-weaning treatments, balanced as much as possible for litter size, location in the farrowing house and the ratio of male/female offspring to ensure not all enriched/barren pens were on one side of the farrowing house or from a certain litter size, and to ensure a balanced male/female piglet ratio. As mentioned earlier, all sows had a hessian rope. In addition, half of the pens were enriched (“Enriched”) with a hessian sack (0.2 m × 0.2 m), a coconut basket (around 0.25 m × 0.2 m), a rubber chewable bone-shaped dog toy (0.25 m), and a bamboo piece (0.3 m), and the other half were kept barren (“Barren,” other than the rope for the sow). The hessian sack and the coconut basket were provided 1 week after birth, the chewable toy in the following week, and the bamboo piece a week thereafter. All enrichment was suspended from the side of the pen (0.1 m above ground) and was replenished if depleted during daily inspection.

Post weaning, all pigs were provided with the same eight enrichment items until slaughter (including an elevated dispenser supplied with fresh-cut grass and seven other items. A list of items provided in the weaner and finisher stage is given in Table 1). These were selected so that they were appropriate to the pigs' age in the weaner and finisher stages. In a previous study (13), a variety of enrichment materials were used for undocked pigs housed on fully slatted floors, and resulted in no tail biting outbreaks, and low levels of tail damage. The most used and sustainable materials in that study (13) were chosen for this study. The materials were categorized based on different properties identified in Van De Weerd et al. (32), and are detailed in Supplementary Table 1. Most items which could be destroyed or ingested were made of organic and biodegradable materials for the health and safety of the pigs. The quantity of the fresh cut grass provided was determined using data gathered in the previous study (13) and varied with the pigs' age (Table 2 and Supplementary Figure 1). In that study (13), we observed a reduction in the use of loose materials in the final weeks during the finisher stage, therefore, the amount of grass was reduced in all treatments to avoid the grass staying in the rack for too long and becoming rotten and unsuitable for use.

**Table 1:** List of enrichment items provided for all pigs during the weaner and finisher stage.

**Item**	**Size**	**Method of provision**
**WEANER**		
2 × Easyfix® Luna 117	Shape as a sphere in the middle with a diameter of 0.12 m and 12 legs (each around 0.12 m long)	Loose on the floor
Spruce (*Picea sitchensis*) post	1.2 m × 0.05 m × 0.04 m	Placed in a dispenser on the wall (the bottom end touching the floor)
Pine (*Pinus sylvestris L*.) block	0.2 m × 0.05 m × 0.05 m	Suspended on a chain
Fresh-cut grass	N/A	Loose in an elevated rack
Cardboard tube	Length around 0.33 m with a diameter around 0.1 m	Suspended on a chain
Rubber pipe	Length around 0.3 m with a diameter around 0.05 m	Suspended on a chain
2 × Ayous (*Triplochiton scleroxylon*) thin sticks	0.15 m × 0.03 m × 0.005 m	Suspended together on a chain
**FINISHER**		
Larch (*Larix decidua*) floor toy	Shape as a squared block in the middle with a perimeter of around 0.27 m and six legs each with a length of around 0.1 m	Loose on the floor
Spruce floor toy	Shape as a squared block in the middle with a perimeter of around 0.3 m and six legs each with a length of around 0.1 m	Loose on the floor
Larch post	1.2 m × 0.08 m × 0.04 m	Placed in a dispenser on the wall (the bottom end touching the floor)
Spruce block	0.33 m × 0.05 m × 0.04 m	Suspended on a chain
Fresh-cut grass	N/A	Loose in an elevated rack
Hessian sack	0.5 m × 0.76 m	Suspended
Easyfix® Astro 200	Four legs (each around 0.2 m long) extending from a central holding point	Suspended on a chain
Bamboo	Around 0.3 m with a diameter of 0.07 m	Suspended on a chain

*A detailed list of the properties for each item is provided in Supplementary Table 1*.

**Table 2:** The quantity of replenishment of fresh-cut grass provided at each check.

	**Weaner**		**Finisher**	
	**Week 1–3**	**Week 4–7**	**Week 1–7**	**Week 8–9**
High	0.5 kg	1 kg	1.5 kg	1 kg
Medium / Low	0.3 kg	0.5 kg	1 kg	0.5 kg

“*High” pigs were checked 3 times per day, “Medium” pigs once each morning, and “Low” pigs 3 times a week (Monday/Wednesday/Friday)*.

The post-weaning enrichment treatment differed in the replenishment rate as follows:

“High” (*ad libitum*): The fresh cut grass was checked 3 times daily (around 0900–1000 h, 1400–1500, and 1800–1900 h) and immediately replenished if depleted, so that it was effectively provided *ad libitum*. All other destructible items were replaced immediately once it was noted during inspection that they were depleted.

“Medium”: The fresh cut grass was replenished with a reduced quantity once daily if depleted and other destructible items were replenished 48 h after depletion.

“Low”: The fresh cut grass was replenished only on Monday/Wednesday/Friday if depleted with the same reduced quantity as “Medium,” and other destructible items were replenished 1 week after depletion.

**Figure 1:**
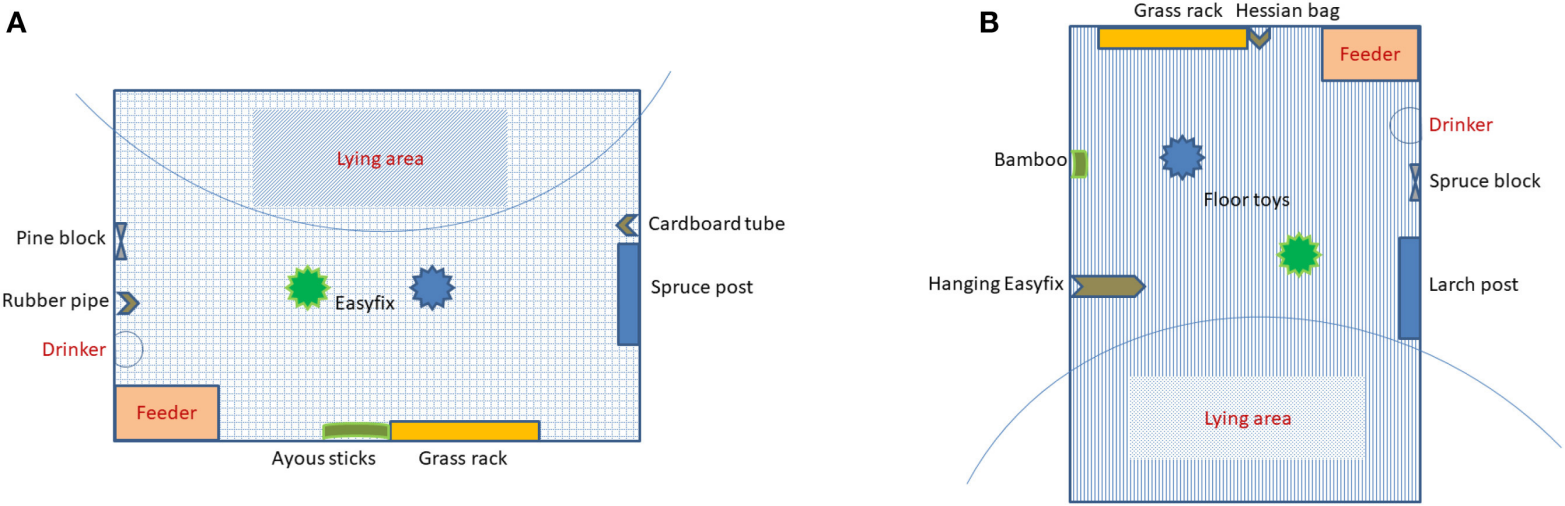
Enrichment layout in the **(A)** weaner pen and **(B)** finisher pen.

A metal wire grid rack (0.59 m × 0.26 m × 0.25 m) was used for dispensing the fresh cut grass. It was fitted on a side of the pen 0.6 m above ground, and 0.8 m from the feeder. The rack wire grid openings measured 2.5 × 2.5 cm (Supplementary Figure 1). The provision of enrichment did not obstruct the slatted-floor area or occupy the pigs' main lying area (Figures 1A,B, and actual photos in Supplementary Figures 2A,B).

### Measurements

All measurements described below were recorded post-weaning.

#### Enrichment

The quantity provided and replacement rate of the fresh-cut grass was recorded. The floor toys in both stages, the spruce and larch post, the spruce block and the hanging toy provided in the finisher stage were weighed at the start and finish of the study (or whenever replaced). The replacement rate of all items was also recorded.

#### Pig Physical Measures

##### Growth

Pigs were weighed individually at weaning (day 0), upon transfer to the finisher house (day 49), 6 weeks into the finishing stage (day 91) and before first batch of slaughter (day 113). Pigs were sent for slaughter in 3-week batches, starting from 20 weeks of age, once reaching 110 kg live weight. This was recorded as the duration of finishing. The daily feed intake was recorded from 1 week after weaning when pigs finished the starter diet.

##### Tail and ear lesions

Tail lesion and ear lesion scores were recorded for individual pigs on a fortnightly basis. Tail lesions were scored using the scoring system developed by the FareWellDock consortium (http://www.farewelldock.eu, 28; tail damage score-−0: no lesion, 1: bite marks, 2: open wound, 3: swollen bite wounds; tail blood score-−0: no blood, 1: black scar, 2: older red blood, 3: fresh blood). The amount of tail amputation was visually estimated and scored on a 0–3 scale; 0: no cannibalism, 1: partly shortened longer than ½ of a normal undocked tail, 2: partly shortened shorter than ½ of a normal undocked tail but longer than ¼ of a normal undocked tail, and 3: shorter than ¼ of a normal undocked tail. Ear lesions were scored using the following system (0: no lesion, 1: superficial scratches, 2: evidence of recent bleeding, 3: substantial cuts and bleeding, 4: part of ear amputated; (17)).

##### Tail posture

During tail lesion scoring sessions, tail posture was also recorded using the protocol developed by Lahrmann et al. (33): 0-up (curled up), 1-down (hanging), 2-tucked (down and tucked into the body).

##### Direct behavior observation

Direct behavior observations were conducted at the pen level using two methods by one observer: all occurrence sampling on pigs' damaging and positive behaviors at the pen level using a prescribed ethogram, and focal sampling on the enrichment (Table 3). The duration of observation for each sampling method was 5 min (i.e., 5 min on pig behaviors and another 5 min focusing on the pigs' interaction with the enrichment). Frequency of behaviors were recorded and a bout of behavior longer than 1 min was counted as a new bout. The observations were carried out on each pen twice every week, with one session in the morning between 1000 h and 1300 h on one day and one session in the afternoon between 1500 h and 1800 h on a different day. Sampling days were distributed across different days of a week so that whenever possible, the same batch of pigs was not always sampled on the same day of the week. During the observation, the number of pigs lying inactive for the whole 5 min was also recorded.

**Table 3:** Ethogram for behavior observation adapted from (13).

**Behaviors**	**Description**
**Enrichment directed behaviors**	
Interact with the item	Any oral manipulation of the items with mouth open, manipulation of, or moving the items using the snout, or any physical contact with the item other than mouth/snout, whether standing, sitting or lying
Aggressive encounter	Biting, head-knocking or pushing over access to the device
**Damaging behaviors**	
Tail manipulation not at feeder (standing or lying/sitting)	Oral manipulation of the tail (tail-in-mouth) of another pig not feeding at the feeder
Tail manipulation at feeder	Oral manipulation of the tail (tail-in-mouth) of another pig which is feeding at the feeder
Ear manipulation (standing or lying/sitting)	Oral manipulation of the ear (ear-in-mouth) of another pig
Biting other parts of the body	Biting a pen mate in another region other than tail and ear, for example, hock, flank, snout, or genital area
Belly nosing	Rubbing/manipulating a pen mate's belly/flank region with rhythmic up and down snout movement
Mounting	Putting two front legs on top of another pig
Aggressive behavior	Pushing, head-knocking and open-mouth fighting with pen mates
**Positive behaviors**	
Social nosing (face)	Gentle, non-open mouth nosing on another pen mate's facial area (without reaction from the recipient) (34)
Individual play	Any scampering, pivoting, head tossing, flopping and pawing movement (35, 36)

##### Post-mortem inspection

Before pigs were sent for slaughter, coded tattoos were applied for individual identification at the slaughterhouse. The carcasses were inspected for tail damage on the processing line after scalding using the system developed by Harley et al. [(4); 0: no lesion, 1: healed/mild lesions, 2: evidence of chewing and puncture wounds, 3: signs of swelling and infection, 4: partial/total loss of tail]. Carcass and visceral condemnation records were obtained from the veterinary inspectors on site. The individual carcass quality report, including cold weight, the percentage of lean meat, muscle (%), and fat (%) data, was obtained from the slaughterhouse.

#### Cost Estimation

The grams used up per pig per day was calculated for the enrichment items whose weight loss data were available (i.e., grass, wooden posts, floor toys, spruce block, and rubber hanging toy). The grass and cardboard tubes used in the study were obtained free of charge and therefore an estimation of the market price was used for the analysis (average market price of grass silage was used in lieu of fresh cut grass since no price for fresh cut grass was available). The costs of items for which weights were unavailable were calculated using the replacement rate, and the estimated cost per piece of item. The pine block, rubber pipe and bamboo were minimally used during the whole study, and therefore the cost was a rough estimation as the equivalence of cost as 10 grams of pine block used, based on a previous study (13). The total enrichment cost was calculated for 48 days in the weaner stage and 64 days in the finisher stage. It is important to note that the cost estimation presented hereafter only consists of the consumable materials used during the entire post-weaning period.

### Statistical Analyses

SAS Base 9.4 (SAS Institute Inc., Cary, NC, USA) was used for data analyses. The weight of enrichment used, the weight gain of the pigs, the pigs' feed intake, behavioral data, and carcass qualities were analyzed using linear mixed models. For the weight of enrichment used, a logarithm transformation was used as residuals were not normally distributed when the raw data were analyzed. Behavior data were analyzed as frequencies per pig per minute, and an arcsine square root transformation was used to transform data to meet the model assumptions. All models included treatment (pre- and post-weaning treatments and their interaction) and batch as fixed effects, and day (for enrichment weight data) or session nested within week (for behavior data) as the repeated effect. For behavioral data, week was also included in the model as a fixed effect and the number of pigs in a pen as a covariate.

The replacement rate of enrichment, the removal of pigs and the duration of finishing were count data and analyzed using a generalized linear mixed model with a Poisson distribution and log link function, and including treatment (pre- and post-weaning treatments and their interaction) and batch as fixed effects.

Lesion scores and tail posture were analyzed as the proportion of pigs at each level of the scoring system in a pen using a linear mixed model, and an arcsine square root transformation was used when residuals were non-normally distributed. If transformations failed to generate a normal distribution for the residuals, generalized linear mixed models were used instead (with a Poisson distribution and log link function). Post-mortem tail scores were also analyzed using a generalized linear mixed model similar to lesion scores recorded alive. The models included treatment (pre- and post-weaning treatments and their interaction) and batch as fixed effects, and for repeated scorings when pigs were alive, week was also included both as both a fixed and repeated effect.

All analytical data are presented as Least Square Mean ± standard error.

## Results

### Enrichment Measurement

**Figure 2:**
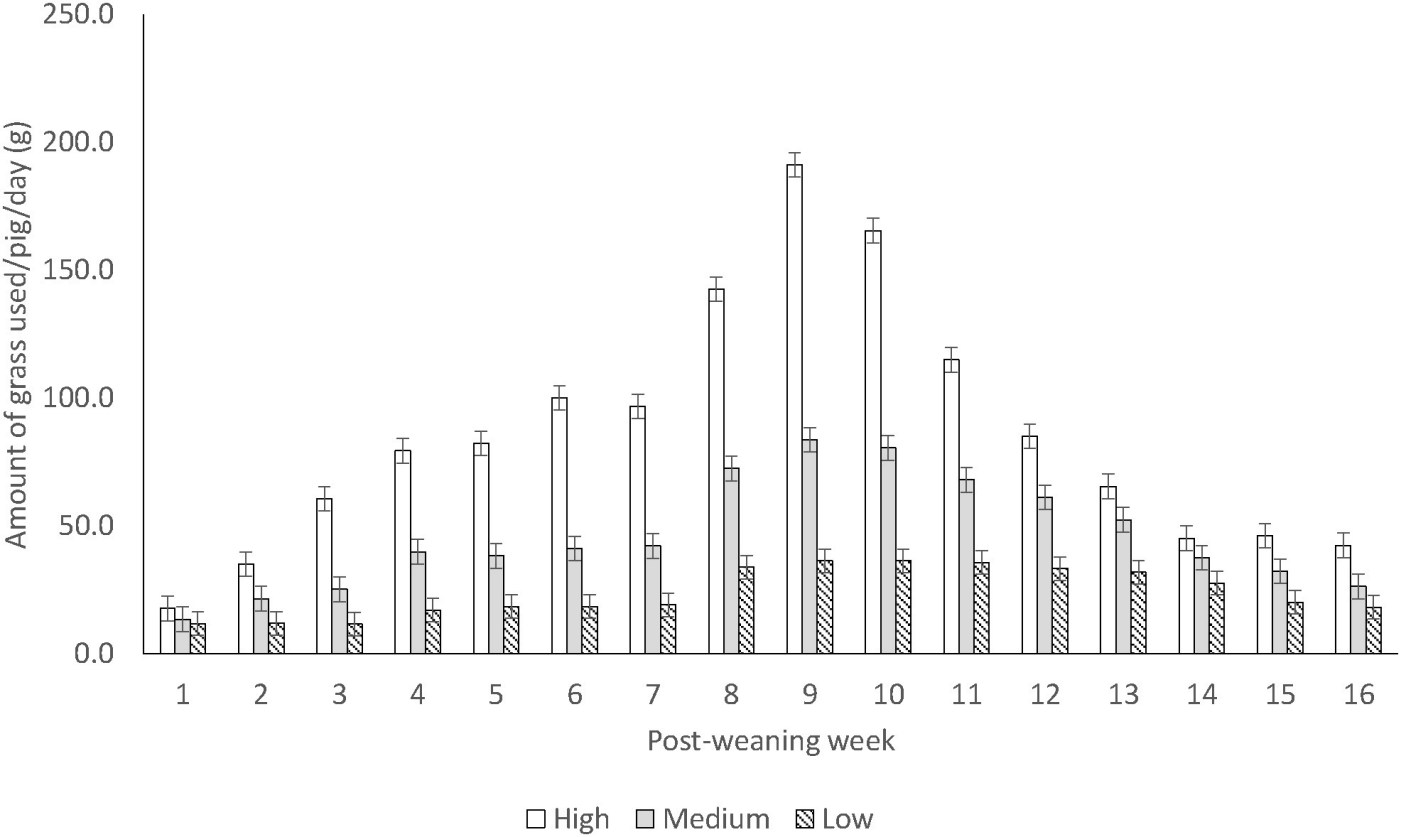
Mean (± s.e.) amount of grass (g/pig/day) used between treatments over weeks post-weaning (*P* < 0.001). Weaner stage was from week 1–7 and finisher stage from week 8–16. “High” pigs were checked/replenished 3 times per day, “Medium” pigs once each morning, and “Low” pigs 3 times a week (Monday/Wednesday/Friday).

In terms of the weight loss of different enrichment materials, there was only an effect of treatment on the quantity of grass. Calculated as the total grass provided per week per pen, “High” enrichment replenishment rate pigs used up more grass than “Medium,” followed by “Low” pigs (“High” 85.7 ± 2.9 vs. “Medium” 46.0 ± 2.9 vs. “Low” 23.9 ± 2.6 g/day/pig; *F*_(38.9, 2)_ = 149.95, *P* < 0.001). If calculated by the time taken for the pigs to use up the grass provided, “High” pigs used up the grass the most quickly, but there was no difference between “Medium” and “Low” pigs (“High” 1.62 ± 0.09 vs. “Medium” 2.32 ± 0.09 and “Low” 2.41 ± 0.08 day/kg; *F*_(37.9, 2)_ = 33.39, both at *P* < 0.001) as “Low” pigs tended to use up all grass within a day. There was also a difference in the interaction between post-weaning treatment and week (Figure 2, *P* < 0.001). In the weaner stage, pre-weaning “Barren” pen pigs tended to use up more of the rubber floor toys than “Enriched” pen pigs (0.029 ± 0.005 vs. 0.017 ± 0.004 g/pig/day; *F*_(32.57, 1)_ = 4.14, *P* = 0.05), but there was no difference in any other item. Neither was there a difference between treatments in the replacement rate of any other enrichment items.

### Growth

Pre-weaning treatment did not have an effect on average daily gain (ADG) in any stage. Weaning weight and the weight at the end of the weaner stage were the same between treatments. Post-weaning “High” pigs had a greater ADG than “Low” pigs during the finisher stage (“High” 1.12 ± 0.01 vs. “Low” 1.08 ± 0.01 kg/day; *F*_(545, 2)_ = 3.18, *P* = 0.04), and the difference was greater during the first 6 weeks than the whole 9 weeks (“High” 1.06 ± 0.01 vs. “Low” 1.01 ± 0.01 kg/day; *F*_(544, 2)_ = 5.4, *P* < 0.01). There was no difference in ADG during the weaner stage, and thus overall, weight gain tended to be higher in “High” than “Low” pigs (*P* = 0.06). No difference was found in average daily feed intake and feed conversion ratio between treatments in the weaner or finisher stage. The finishing duration did not differ between treatments.

### Lesions and Tail Posture

There was no effect of pre- or post-weaning treatment on tail lesion scores either in terms of damage or blood scores. For tail postures, 93.44 ± 0.51% of pigs showed curled-up tails, but pre-weaning “Enriched” pigs had a lower proportion of individuals showing down and tucked tails compared to “Barren” pigs (0.04 ± 0.01 vs. 0.07 ± 0.01; *F*_(50.41, 1)_ = 4.67, *P* = 0.04).

For ear lesions, pre-weaning “Enriched” pigs had a higher proportion of score 0 than “Barren” pigs (0.071 ± 0.006 vs. 0.056 ± 0.006; *F*_(38.6, 1)_ = 4.2, *P* = 0.04).

No difference in tail amputation score was found between treatments. At the end of the study, 72.57% of pigs had intact tails without amputation, 23.44% had tail amputation score 1 (meaning that more than half the tail remained), 0.69% had moderate amputated tails (score 2; less than half the tail remained) and no pigs scored 3.

### Behavior

There was no effect of pre-weaning treatment on any of the behaviors observed. After weaning, “High” pigs performed less damaging behavior (tail/ear biting, other biting, belly-nosing, mounting and aggressive behavior) than “Low” pigs (0.0101 ± 0.0004 vs. 0.0120 ± 0.0004, *F*_(38, 2)_ = 5.14, *P* = 0.01). No other differences in behavioral measures were found between post-weaning treatments.

**Figure 3:**
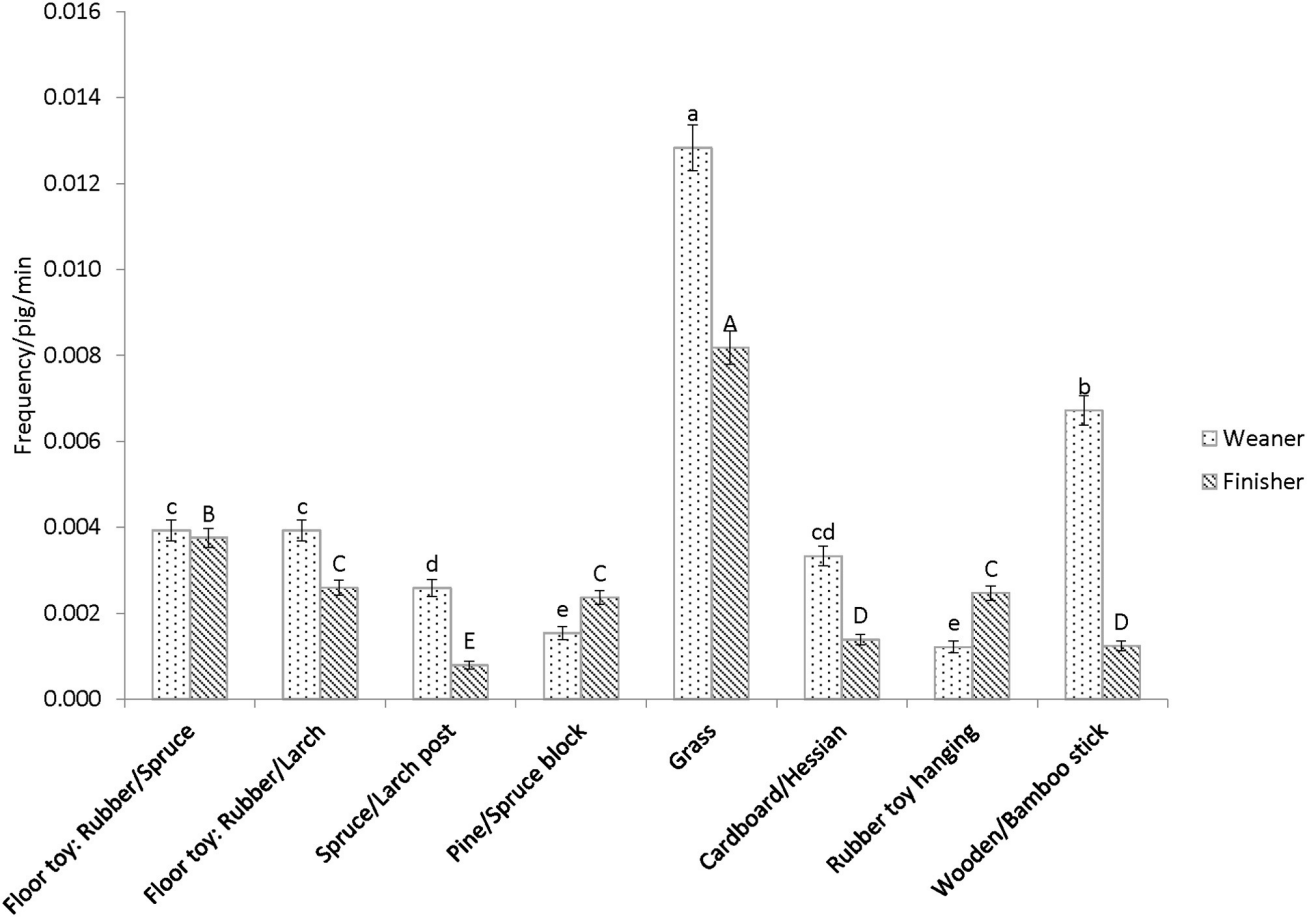
Frequency of interaction by pigs with different items in the weaner and finisher stage. Different small letters denote differences between items in the weaner stage (*P* < 0.001), and capital letter for the finisher stage (*P* < 0.001), after Tukey-Kramer adjustment.

**Figure 4:**
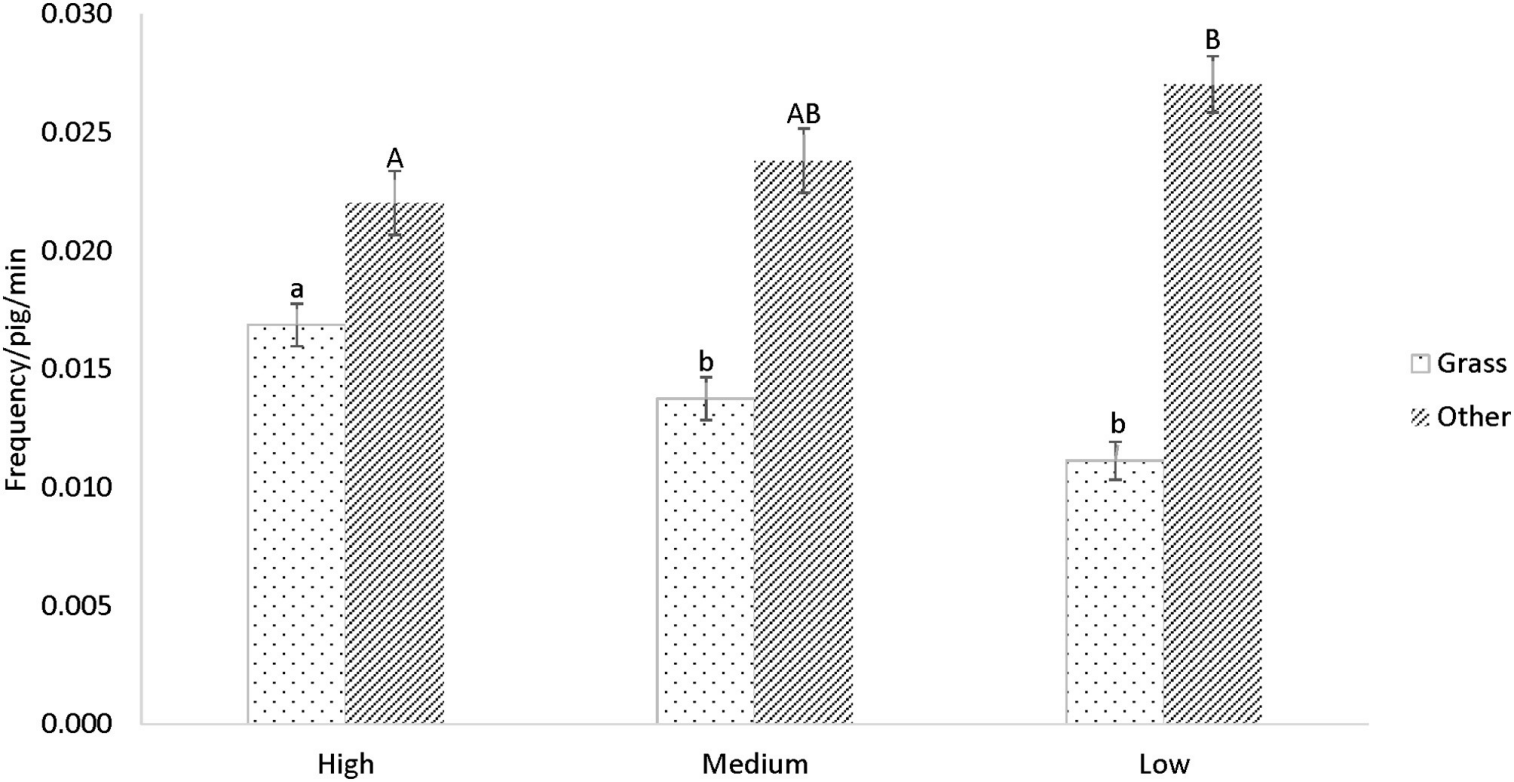
Mean (± s.e.) frequency of interaction with grass and all other items between treatments in the weaner stage. Different small letters denote differences in grass interaction (*P* < 0.001), and capital letters denote differences in interactions with other items between treatments (*P* = 0.02). Differences were indicated after Tukey-Kramer adjustment.

The overall amount of interaction with all enrichment items combined did not differ between treatments, but pigs exhibited a preference for different items, and similar preferences were found in both the weaner and finisher stage (Figure 3). Grass was the most preferred enrichment item; during the weaner stage, more interactions with grass were observed in “High” than “Medium” or “Low” pigs (*P* < 0.001, Figure 4). Conversely, the total interaction with all items other than grass was greater in “Low” than “High” groups (*P* = 0.02, Figure 4) in the weaner stage. The difference did not extend into the finisher stage.

**Figure 5:**
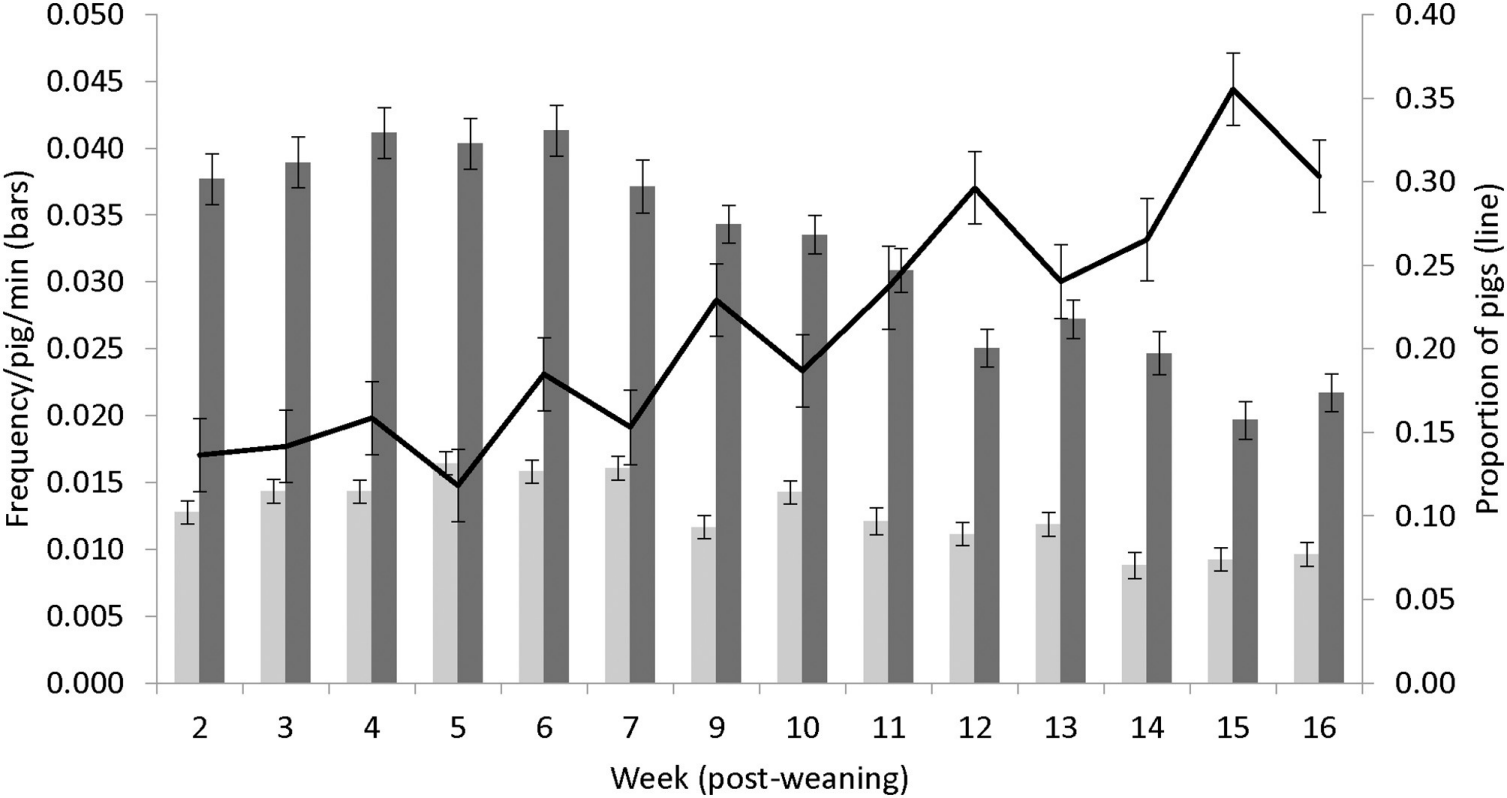
Mean (± s.e.) activity level over time based on (A) all behaviors combined (damaging + positive behaviors, see Table 3 for full ethogram, light gray bars, as frequency/pig/min) (B) total enrichment interactions (dark gray bars, as frequency/pig/min) and (C) proportion of pigs lying inactively (black line).

Both pen level behaviors and the amount of interaction with the enrichment showed a gradual declining trend over time, whereas the proportion of pigs lying inactive increased, as the pigs grew older (Figure 5). The total amount of behaviors observed and the proportion of pigs inactive did not differ between treatments.

### Post-mortem Examinations

No difference was found between treatments in any of the post-mortem measures (i.e. tail lesions, level of tail amputation, the presence or absence of tail amputation, cold weight, and the percentage of lean meat, muscle, and fat).

### Severity of Tail Biting Events

In total, 76 pigs were temporarily removed as tail biting victims (18 from “High” replenishment pens, 39 from “Medium” pens and 19 from “Low” pens; six pigs were removed twice and one pig was removed 3 times). Twenty-two pigs were removed as tail biters (6 from “High” pens and 16 from “Medium” pens), and 4 pigs had antibiotic injections in their home pen due to being tail bitten. There were 23 pigs removed from the study prematurely, out of which 2 pigs needed to be euthanised due to whole body and hind leg paralyzes that may have been associated with tail biting. Others were removed due to health issues, including one case of tail necrosis in a pen without any history of tail biting, hernia (seven pigs), respiratory failure (one pig), lameness (two pigs), suspected meningitis (two pigs) and unknown sudden deaths (eight pigs). The removals and health issues of the pigs were not affected by the treatments. There were 14 tail biting outbreaks from 12 pens (with two pens had recurring outbreaks; one in “Barren”-“Medium” and one in “Enriched”-“High”) and no difference was found between treatments (four outbreaks in “High” pens, five in “Medium” pens and three in “Low” pens).

### Cost Estimation

The estimated cost of each item in each post-weaning treatment is presented in Supplementary Table 2. The overall enrichment cost was numerically higher in “Medium,” followed by “Low” and “High.” The highest enrichment cost in all three treatments was the wooden floor toys, followed by grass in “High” and “Medium” pens.

## Discussion

This study investigated how both pre-weaning exposure to enrichment, and different post-weaning enrichment replenishment strategies, could influence the performance of tail biting and productivity of undocked pigs in fully slatted systems. To the best of our knowledge, this is a first attempt to use the same multiple point-source enrichment items, but varying replacement rates to create a range of treatments, to investigate these outcomes.

Although some tail biting occurred in all treatments, both the severity and occurrence of biting was substantially reduced compared to our earlier study on the same farm which had used only one enrichment item per pen (37). However, it was somewhat higher than another, smaller study we performed, with multiple enrichment items provided per pen, on an *ad libitum* basis (i.e. all replenished as soon as they were exhausted; (13)). No difference in lesion scores was found between post-weaning treatments, but pigs in the enriched pre-weaning environment had slightly lower ear lesion scores. Pigs with a “Low” enrichment replacement rate post-weaning performed more damaging behaviors, but did not experience more tail biting outbreaks compared to “High” and “Medium.” Thus, provision of multiple types of enrichment regardless of replenishment rate was effective in reducing the amount of severe tail biting incidents. A more frequent replenishment of materials as soon as they were depleted, could bring further advantage in generating pigs' sustained interest, reducing damaging behaviors and also promoting pigs' growth in the long term.

### Effect of Pre-weaning Exposure to Enrichment

Whether or not pigs were enriched in the pre-weaning stage did not influence ADG at any stage. Other studies have also shown that post-weaning enrichment had a greater effect on the weight gain of pigs than early life experience (25, 38, 39). Brajon et al. (40) found pre-weaning enrichment provision only increased weaners' weight gain immediately post-weaning, which again suggests a transient effect compared to post-weaning treatments.

Pre-weaning exposure to enrichment only contributed to a slight reduction in both ear lesion scores and downward tail postures. Ear lesions can be caused by ear biting or ear necrosis, but still very little is understood regarding their causes and risk factors (41). Although no difference in ear biting behavior was found between pre-weaning treatments in the current study, it is possible that pre-weaning enriched pigs may have been more accustomed to biting enrichment and therefore less likely to bite ears at early stages post-weaning, when ear necrosis is commonly reported to develop (41). As the etiology of ear necrosis involves establishment of bacteria in wounds of the ear, as well as physical damage resulting in broken skin, even a low level of difference in ear biting could have resulted in differential development of lesions (41).

Downward tail postures are recognized as a reaction to tail-directed behaviors (33, 42). However, although “Enriched” pigs exhibited fewer tucked tails scored over the course of the study, there was no demonstrable effect of pre-weaning treatment on tail lesions or tail biting behaviors. Tail posture has been found to be associated with emotional state and a curly tail is considered as the undisturbed default position without the effect of external stimuli, when pigs are reared in a good welfare environment (43, 44). It is possible that tail posture scored inside the pen may be more affected by the disturbance of human presence than the status of the pigs at the time of scoring, and thus be less reliable as an indicator of tail damage.

It is important to note that since the farrowing pens had fully slatted floors, the enrichment items provided to the piglets were all suspended, and bedding or substrates suitable for provision on the floor were not presented to the pigs. This may be the reason why only a limited number of effects were found, contrary to the positive effects reported in other studies which used loose substrates as enrichment during the suckling stage (25, 38, 45). However, Oostindjer et al. (27) found no effect of pre-weaning provision of wood shavings, peat and straw on pig's post-weaning manipulative behaviors, but a more influential factor was whether the sow was confined or loose. This suggests that the influence of early life experience on the development of damaging behaviors may entail more than just environmental enrichment. Social environment such as piglet-sow interactions, and inter-litter socialization, should also be considered, as studies have also suggested early socialization reduces aggression at mixing (46), and thus could help to reduce overall stress levels. Based on the current results, the effect of early exposure to point-source enrichment appeared to only have a minor effect on pigs later in life.

### Effect of Post-weaning Enrichment Management

#### Enrichment Use

Calculated on a “per-week” basis, “High” pigs used up the most grass post-weaning, followed by “Medium” and then “Low” pigs. This is not surprising as the main difference between these treatments was the different quantities and replenishment rates of grass; therefore, pigs which were provided with more grass used up more grass. There is scant research reporting on the amount and replenishment rate of loose enrichment materials used, and therefore it is difficult to compare our results with others. Previously, only the quantity of straw required to provide permanent access to pigs (*ad libitum*) was quantified (47). However, we did not record the amount of grass left in the rack and thus could not conclude the amount of grass we provided in “High” pens indeed saturated pigs' needs with regard to access to a loose edible material.

On the other hand, when calculated by how fast the pigs used up the grass provided to them, “High” pigs were also the fastest, but “Medium” pigs were not faster than “Low” pigs. Although the replenishment strategy for “Low” pigs was 3 times per week, “Low” pigs did not usually take 2 days or more to use up the grass, but rather emptied the rack within a day. This demonstrated that the grass provided was a biologically relevant resource for pigs, as when providing in a small ration, pigs used it up equally quickly regardless of receiving it daily or 3 days per week. From the management's perspective, a daily routine may also be easier to incorporate into habitual practice than on 3 days per week.

Similarly, the behavior observation also confirmed that pigs valued grass the most and showed a preference to the other enrichment materials provided. This result agrees with other studies where similar loose materials were preferred over point-source items (13, 23, 32, 48). “High” pigs used up a higher quantity of grass than the other treatments and interacted the most with grass in the weaner stage. In a study which compared a different number of racks (containing the same amount of chopped straw), greater levels of interaction were also found in groups with more racks, that is, more straw available at the same time (15). Thus, it appears that the greater the quantity of loose material in the pen, the greater the amount of interaction there is with it. In contrast, “Low” pigs interacted with all items other than grass more than “High” pigs. This may be due to the fact that the grass rack was more likely to be empty in “Low” pens. Scott et al. (49) found that when a hanging toy was provided in a barren environment, it attracted more interactions from the pigs than in a straw-bedded pen. When the more preferred resource is absent, pigs might divert their attention to less favorable items. On the other hand, if point-source items possess properties such as being chewable and ingestible, they can be attractive to pigs even in the presence of more favored items (32). Therefore, provision of a variety of biologically relevant enrichment items as well as loose materials could encourage more interactions from pigs when the loose material is depleted. It would also be interesting to further explore the individual variation in pig's enrichment use, when there are multiple biologically meaningful enrichment present. In the current study, the grass dispenser is a long rack, so there could be multiple pigs using grass at the same time, but future research could investigate whether pig's social hierarchy influences their access to enrichment compared to that of the feeder.

Other than grass, weaners interacted most with hanging ayous sticks, followed by the rubber floor toy, spruce post and hanging cardboard tube. The finishers showed a similar preference for the wooden floor toys, followed by the hanging spruce block and rubber toy. Point-source items are generally thought to be more preferred by pigs when suspended compared to when loose on the floor, due to difficulties in maintaining cleanliness when on the floor (22). However, in the current study, this was evidently not the case. The floor toys used were designed to prevent them from being soiled easily, and thus their attractiveness was not hampered by a lack of hygiene. If hygiene standards are good, and the items on the floor have some properties that pigs prefer (e.g. being destructible, deformable or chewable), then they could be favorable to pigs, as they facilitate a head down, rooting action, which is part of their natural behavior repertoire.

Nevertheless, the interaction with enrichment materials declined over time. This is likely due to the fact that pigs became less active in general as they aged, as shown in the proportion of pigs observed lying inactively. Since we did not observe a sharp drop in inactivity level when they moved into the finisher accommodation, where the enrichment items provided were refreshed and novel, and there was more space available inside the pen, the reduced interaction with enrichment could not be due to pigs being accustomed to the enrichment items or a lack of space.

#### Growth and Carcass Quality

There was an increase in ADG in the finisher stage when pigs received a high rate of enrichment replenishment compared to the low treatment, albeit with no difference between treatments in terms of feed efficiency. The positive effect of post-weaning straw provision on feed efficiency has been reviewed extensively (23, 50–52). However, point-source items and substrates provided via dispensers did not seem to affect growth in previous studies (22, 23). The combination of loose substrates in dispensers, along with numerous other items that are biologically relevant for pigs, could have enhanced the positive effect of enrichment to stimulate growth in the current study. Similar to what we found, Holinger et al. (53) reported that when pigs were fed grass silage, there was no effect on the time to slaughter, slaughter weight or carcass traits. However, they found grass silage reduced the prevalence of gastric ulcers in pigs. It is possible that “High” pigs in our study had improved gastric health and therefore weight gain, although without post-mortem examinations, it is not conclusive why the high enrichment replenishment improved ADG in pigs. Further research is needed to explore the benefit of fresh grass to pigs' gastric health and its possible contribution to other growth parameters.

Although there was no difference in finishing duration between treatments, 50% of the pigs reached slaughter weight (114.47 ± 0.48 kg) at 20 weeks of age, which is much shorter than the average finishing duration in Ireland, which is around 25 weeks of age (54). The shorter production period has positive implications for reducing production costs, as there is less occupancy of buildings and feed consumption (55). No difference was found in carcass quality between treatments, which is similar to what previous studies have reported (9, 23). The higher finisher weight gain in “High” pigs and the overall shorter time required to reach slaughter weight both highlight that providing sufficient quantity and quality of environmental enrichment may not only improve pig welfare but also promote pig performance.

#### Physical Scores and the Level of Damaging Behavior

The lesion scores did not differ between post-weaning treatments. However, the observed performance of more damaging behaviors in “Low” than “High” pigs could be related to the low rate of replenishment; they received less grass and sometimes had fewer enrichment items available in the pen as items were not replenished immediately. The lack of agreement between the results from the lesion scoring and the behavior observations could be an anomaly; the duration of observation for each pen was relatively low (10 min/day), so it is possible that it was not sufficient to observe real differences. However, it is not without precedent that differences in damaging behavior are not reflected in lesion scores, and vice versa (13, 56, 57). One reason is that statistical differences in the amount of damaging behavior may not be biologically relevant (i.e. there may be a “threshold” above which physical damage occurs). Indeed, the results are in line with our hypothesis, and with what little previous and similar research we were able to source (i.e. comparing amount provided of the same type(s) of enrichment across all treatments). Most of the published work which has used this strategy has compared different quantities of loose straw. Even so, Day et al. (45) found that performance of damaging behaviors was only different between pigs with or without straw, but not between different quantities given. However, another study found a linear decrease in oral manipulation toward pen mates in relation to the amount of uncut straw provided (47). Although it is difficult to compare provision of loose straw on the floor with the elevated grass dispenser used in this study, the results suggest that the quantity, as much of the type of enrichment provided, can affect the performance of damaging behaviors. Indeed in terms of point-source enrichment items, a meta-analysis suggested that when these are provided in an adequate quality and quantity, negative social behaviors in pigs can be reduced (22).

In a previous study conducted in the same facility by the same experimenter with identical management practices (37), we recorded a high level of tail biting when the pigs only received a single enrichment item with a higher stocking density (Table 4). In contrast, the negative effects of tail biting were reduced in the current study in terms of the number of tail biting outbreaks, the length of outbreaks, the amount of pigs that needed to be temporarily removed due to tail biting events, and the level of tail amputation in pigs (Table 4). No pig had a severe tail amputation. Although sporadic tail biting still occurred, and 14 tail biting outbreaks were recorded, all but two pigs removed temporarily for outbreak control were reintroduced back to their home pens successfully. This demonstrates by using slat-compatible enrichment with a stocking density that is not drastically different from the EU regulation (0.52 m^2^ for growers up to around 30–50 kg and 0.8 m^2^ for finishers up to around 110 kg, c.f. EU minimum space requirement at 0.4 and 0.65 m^2^ respectively), the risk of tail biting in undocked pigs can be reduced.

**Table 4:** Descriptive comparison between the current study and two previous studies (13, 37) conducted by the same authors in the same facility.

	**Chou et al. (37)**	**Chou et al. (13)**	**Current study**
Experimental treatment	Single enrichment (wood or toy) and dietary fiber (high or standard)	Post-weaning same multiple enrichment items throughout or varied	Pre-weaning enrichment or not and post-weaning different enrichment replenishment rate
Pigs used	672	96	576
Stocking density (m^2^/pig)	0.45 m^2^ (growers up to 30–50 kg) 0.69 m^2^ (finishers up to 110 kg)	0.52 m^2^ (growers up to 30–50 kg) 0.8 m^2^ (finishers up to 110 kg)	0.52 m^2^ (growers up to 30–50 kg) 0.8 m^2^ (finishers up to 110 kg)
Number of pigs permanently removed due to tail biting	9	1	2
Number of pigs temporarily removed due to tail biting incidents	252	0	98
Number of pigs receiving antibiotic injections in the home pen due to tail injuries	52	0	4
Number of tail biting outbreaks	26	0	14
Average length of outbreak	19.6	-	13.3
Percentage of pigs without tail amputation at the end of the study	33.1%	99.0%	72.6%

*All studies used undocked pigs*.

### Cost Estimation

The “High” replenishment strategy did not result in the highest enrichment cost overall, partly due to a higher use of grass and a lower replacement rate of other consumable items, which might be more expensive per unit than the estimation of grass used in the current study. The floor toys used in the study were more expensive per unit and replaced numerically more frequently for “Medium” and “Low” pigs, and therefore, the overall cost in these groups increased. Unfortunately the actual time and cost of labor involved in managing the enrichment items was unable to be ascertained since the experimenter who was responsible for checking and replenishing the enrichment was often taking experimental measurements at the same time. Hence, the overall cost could be underestimated as the labor cost is also another important element in cost analysis. However, the cost per production cycle for all treatments did not exceed €2 per pig, which showed that using materials easily available in the local context, for example, grass crops in Ireland, can help reduce cost. The cost of enrichment could also be partly offset by a shorter finishing duration.

## Conclusions

Early exposure to point-source enrichment items in the pre-weaning stage did not exert a strong influence on pigs' later life performance or damaging behaviors, compared to post-weaning enrichment provision. A high rate of enrichment replenishment post weaning did not affect the tail lesions scored, however, it did reduce the occurrence of damaging behaviors observed and improved growth rate in the finisher stage, and the overall cost of enrichment materials was not higher compared with the lower rates of replenishment. This study suggests that it is possible to find a practical and feasible way to keep tail biting in undocked pigs on fully slatted floors at a manageable level by using an enhanced enrichment strategy which includes a good quantity and quality of point-source enrichment items.

## Data Availability Statement

The raw data supporting the conclusions of this article will be made available by the authors, without undue reservation.

## Ethics Statement

The animal study was reviewed and approved by Teagasc Animal Ethics Committee (TAEC163-2017).

## Author Contributions

J-YC: conducting experimental work, data collection, data management, data analysis, and drafting of manuscript. DS, RD'E, and KO'D: project coordination, supervision, manuscript revision, and editing. All authors contributed to the conceptualization, experimental design, reading, and approval of the final manuscript.

## Conflict of Interest

The authors declare that the research was conducted in the absence of any commercial or financial relationships that could be construed as a potential conflict of interest.
