# Supplementary material for: A High Enrichment Replenishment Rate Reduces Damaging Behaviors and Increases Growth Rate in Undocked Pigs Kept in Fully Slatted Pens
Source: Front Vet Sci. 2020 Nov 13;7:584706. doi: 10.3389/fvets.2020.584706 (PMC7691579; doi:10.3389/fvets.2020.584706)
Supplement: Supplementary file 1 [file Data_Sheet_1.pdf]

Supplementary Material

A high enrichment replenishment rate reduces damaging behaviours and increases growth rate in undocked pigs kept in fully slatted pens

Jen-Yun Chou[1,2,3], Dale A. Sandercock[2], Rick B. D'Eath[2*], Keelin O'Driscoll[1]

*** Correspondence:**
Dr Jen-Yun Chou
jenyun.chou@gmail.com

Supplementary Figure 1, 2A and 2B

Supplementary Table 1 and 2

Supplementary Figure 1. Different quantities of grass provided in the rack post weaning

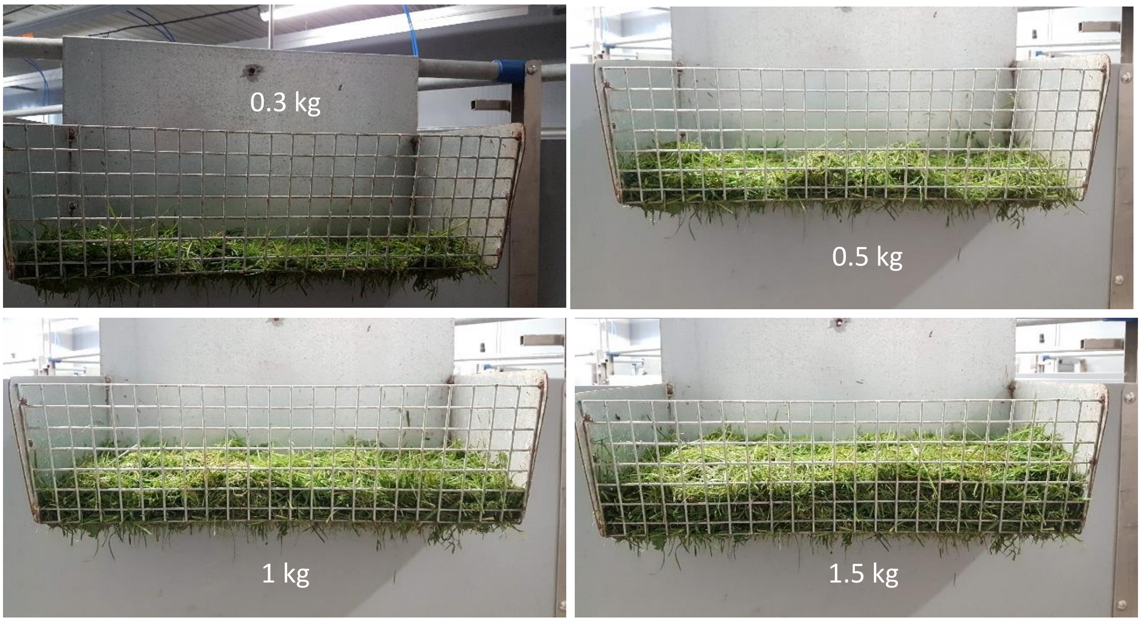

Supplementary Figure 2. Photos of enrichment items provided in the weaner (**A**) and finisher (**B**) stage

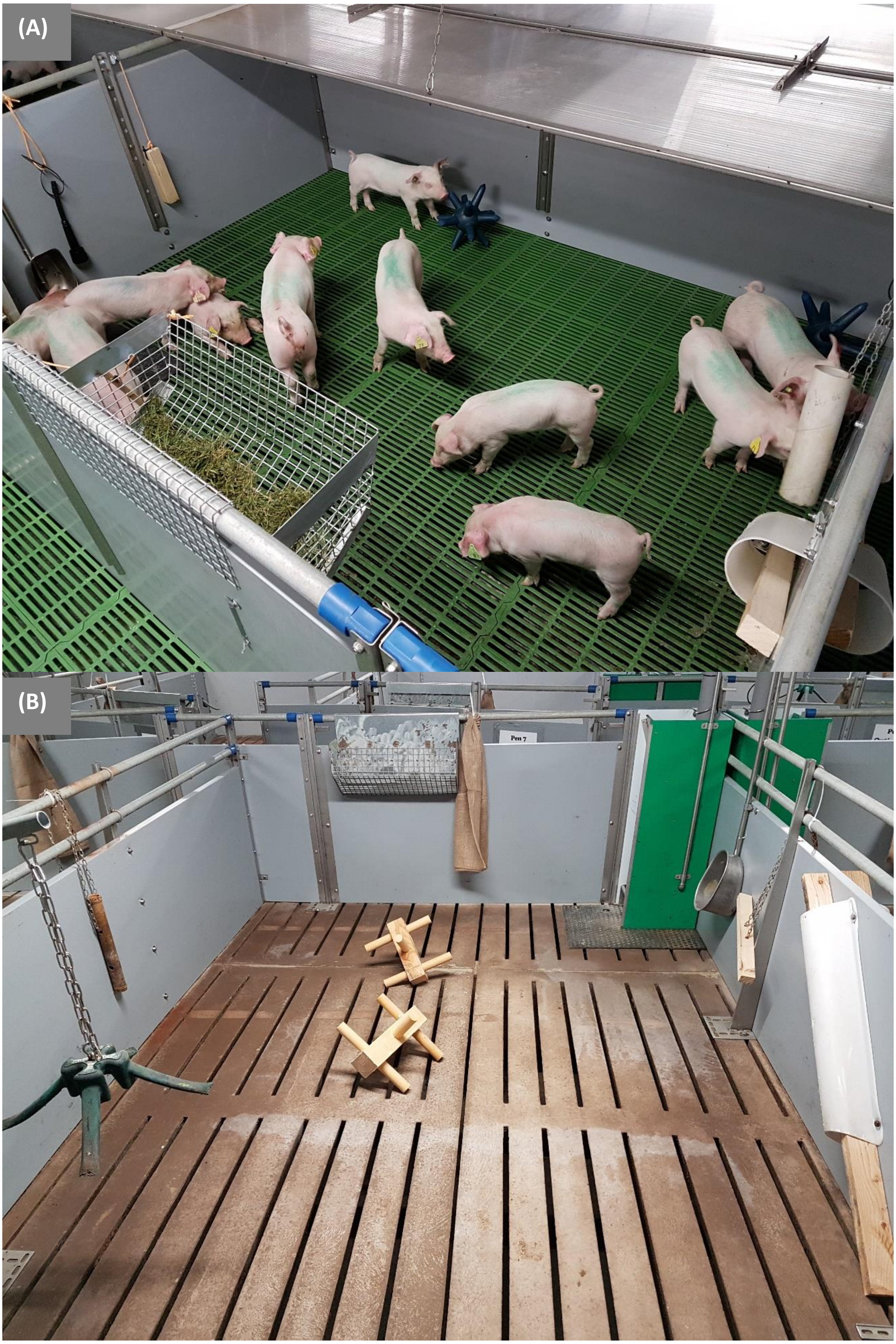

Supplementary Table 1. Enrichment provision list, each with different properties identified in van de Weered et al. (2003).

Stage	Properties of enrichment						Item	Method of provision
	Rootability	Durability	Edibility	Texture	Presentation	Location		
Weaner	Rootable	Deformable	Chewable	soft	Moveable	Floor level	Rubber floor toy (Easyfix® Luna 117)	Loose on the floor
	Rootable	Deformable	Chewable	soft	Moveable	Floor level	Rubber floor toy (Easyfix® Luna 117)	Loose on the floor
	Rootable	Destructible	Ingestible	hard	Attached	Floor level	Spruce post	Placed in a dispenser
	non-rootable	Destructible	Ingestible	hard	suspended	Eye level	Pine block	Suspended on a chain
	non-rootable	Renewed	Ingestible	loose	Attached	Eye level	Fresh-cut grass	In an elevated rack
	non-rootable	Destructible	Chewable	soft	suspended	Eye level	Cardboard tube	Suspended on a chain
	non-rootable	Deformable	Chewable	soft	suspended	Eye level	Rubber pipe	Suspended on a chain
	non-rootable	Destructible	Chewable	hard	suspended	Eye level	2 Ayous sticks	Suspended on a chain
Finisher	Rootable	Destructible	Chewable	hard	Moveable	Floor level	Larch floor toy	Loose on floor
	Rootable	Destructible	Chewable	hard	Moveable	Floor level	Spruce floor toy	Loose on floor
	Rootable	Destructible	Ingestible	hard	Attached	Floor level	Larch post	Placed in dispenser
	non-rootable	Destructible	Ingestible	hard	suspended	Eye level	Spruce block	Suspended on chain
	non-rootable	Renewed	Ingestible	loose	Attached	Eye level	Fresh-cut grass	In an elevated rack
	non-rootable	Destructible	Chewable	soft	suspended	Eye level	Hessian sack	Suspended
	non-rootable	Deformable	Chewable	soft	suspended	Eye level	Rubber hanging toy (Easyfix® Astro 200)	Suspended on a chain
	non-rootable	Destructible	Chewable	hard	suspended	Eye level	Bamboo	Suspended on a chain

Supplementary Table 2. Estimated cost of all enrichment items used in the study

	Unit cost (€)	Enrichment replenishment rate								
		High			Medium			Low		
		Use[1]	Cost/pig/day (€)[1]	Cost/pig/cycle (€)	Use	Cost (€)	Cost/pig/cycle (€)	Use	Cost (€)	Cost/pig/cycle (€)
Grass (g)[2]	0.00003	85.547†	0.003†	0.287	45.315†	0.002†	0.152	23.289†	0.002†	0.078
**Weaner**										
Rubber floor toy (g)	0.016	0.035†	0.0006†	0.0268	0.041†	0.0007†	0.0313	0.036†	0.0006†	0.0275
Spruce post (g)	0.001	0.188†	0.0003†	0.0126	0.246†	0.0003†	0.0166	0.295†	0.0004†	0.0198
Cardboard tube (piece)[3]	0.124	0.417	-	0.052	0.385	-	0.048	0.292	-	0.036
Ayous sticks (2 pieces)	0.732	0.172	-	0.126	0.151	-	0.111	0.151	-	0.111
Pine block (use)[4]	0.015	0.083	-	0.001	0.083	-	0.001	0.083	-	0.001
Rubber pipe (use)[4]	0.015	0.083	-	0.001	0.083	-	0.001	0.083	-	0.001
**Finisher**										
Larch floor toy (g)	0.015	0.400†	0.006†	0.395	0.473†	0.007†	0.466	0.415†	0.006†	0.409
Spruce floor toy (g)	0.015	0.489†	0.007†	0.473	1.037†	0.016†	1.002	1.048†	0.016†	1.012
Larch post (g)	0.001	0.204†	0.0002†	0.012	0.189†	0.0002†	0.011	0.168†	0.0002†	0.010
Spruce block (g)	0.001	0.318†	0.0003†	0.020	0.406†	0.0004†	0.026	0.547†	0.0005†	0.035
Rubber hanging toy (g)	0.022	0.051†	0.001†	0.073	0.006†	0.0001†	0.009	0.025†	0.001†	0.036
Hessian sack (piece)	1.000	0.115	-	0.115	0.089	-	0.089	0.109	-	0.109
Bamboo (piece)[5]	0.254	0.115	-	0.029	0.161	-	0.041	0.115	-	0.029
Total (production cycle)[6]				**1.623**			**2.004**			**1.915**

[1] For all items that were recorded by weight loss (g), the unit for use was g/pig/day (cost: €/pig/day, indicated with †), and for all other items the unit was per pig per weaner or finisher stage. There were two rubber floor toys provided in the weaner pen, so the use and cost were multiplied by two.

[2] The grass in the current study was collected free of charge, so the estimation used the current average grass silage price at €30/tonne. Per cycle cost was calculated based on 112 days in the full production cycle.

[3] The cardboard tubes in the current study were collected free of charge, so the estimation used the market price of similar products.

[4] The pine block and rubber pipe were minimally consumed, and the weight loss is not available. The rough estimation is based on the cost of 10g of the pine block consumed per use per batch. The bamboo was obtained for free and the surrogate cost was unattainable and therefore the same estimation as the pine block was used.

[5] The bamboos in the current study were collected free of charge, so the estimation used the cost of a pine block which was of similar size

[6] The total enrichment cost per cycle was calculated based on 48 days in the weaner stage and 64 days in the finisher stage.
